# Supplementary material for: Bulk-suppressed and surface-sensitive Raman scattering by transferable plasmonic membranes with irregular slot-shaped nanopores
Source: Nat Commun. 2024 Jun 19;15:5236. doi: 10.1038/s41467-024-49130-2 (PMC11187206; doi:10.1038/s41467-024-49130-2)
Supplement: Supplementary file 1 — Supplementary Information [file 41467_2024_49130_MOESM1_ESM.pdf]

Supplementary Information for: Bulk-suppressed and surface-sensitive Raman scattering by transferable plasmonic membranes with irregular slot-shaped nanopores

Roman M. Wyss,[1,2] Günter Kewes,[1] Pietro Marabotti,[1] Stefan M. Koepfli,[3] Karl-Philipp Schlichting,[4] Markus Parzefall,[5] Eric Bonvin,[5] Martin F. Sarott,[6] Morgan Trassin,[6] Maximilian Oezkent,[7] Chen-Hsun Lu,[7] Kevin-P. Gradwohl,[7] Thomas Perrault,[8] Lala Habibova,[1] Giorgia Marcelli,[1] Marcela Giraldo,[6] Jan Vermant,[2] Lukas Novotny,[5] Martin Frimmer,[5] Mads C. Weber,[8] and Sebastian Heeg[1,9]

[1]*Institut für Physik und IRIS Adlershof, Humboldt-Universität zu Berlin, 12489 Berlin, Germany*
[2]*Soft Materials, Department of Materials, ETH Zürich, 8093 Zürich, Switzerland*
[3]*Institute of Electromagnetic Fields (IEF), ETH Zürich, 8092 Zürich, Switzerland*
[4]*Laboratory of Thermodynamics in Emerging Technologies Department of Mechanical and Process Engineering, ETH Zürich, 8092 Zürich, Switzerland*
[5]*Photonics Lab, ETH Zürich, 8093 Zürich, Switzerland*
[6]*Department of Materials, ETH Zürich, 8093 Zürich, Switzerland*
[7]*Leibniz-Institut für Kristallzüchtung, 12489 Berlin, Germany*
[8]*Institut des Molécules et Matériaux du Mans, UMR 6283 CNRS, Le Mans Université, 72085 Le Mans, France*
[9]email: sebastian.heeg@physik.hu-berlin.de

CONTENTS

Suppl. Note 1. Additional Information about PAuM transfer, Pore size distribution and Removal of PAuM from substrate 2

Suppl. Note 2. Interference effects on surface enhancement 5

Suppl. Note 3. Interference effects on bulk suppression 6

Suppl. Note 4. Numerical Finite Element Simulations 8

Suppl. Note 5. Depth-dependence of surface Raman enhancement 11

Suppl. Note 6. Graphene 2D-mode enhancement with Spacers 13

Suppl. Note 7. PAuM-enhanced Raman spectroscopy on $LaNiO_3$ thin films 16

Suppl. Note 8. Surface-sensitive Raman scattering at low temperatures 17

Suppl. Note 9. Feasibility of using PAuM to probe rough surfaces 17

References 18

Supplementary References 18

SUPPL. NOTE 1. ADDITIONAL INFORMATION ABOUT PAUM TRANSFER, PORE SIZE DISTRIBUTION AND REMOVAL OF PAUM FROM SUBSTRATE

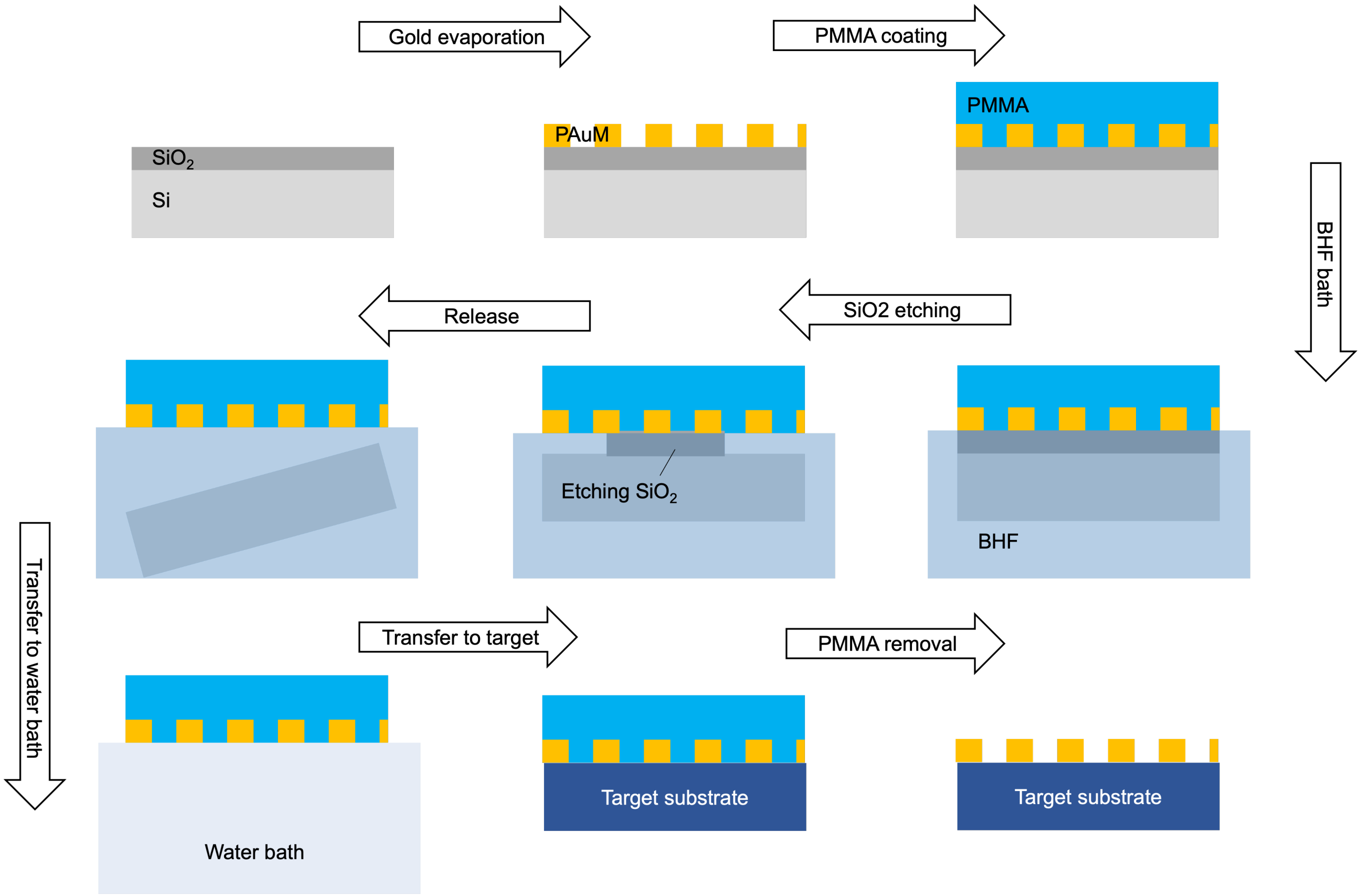

SUPPLEMENTARY FIGURE 1. **Manufacturing and transfer process for a PAuM to an arbitrary substrate.** Note that depending on the nature of the target substrate (graphene, oxide etc.) the PMMA is either removed by a series of solvent baths (acetone, isopropyl alcohol) or oxygen plasma, such that the target substrate is not damaged. BHF: buffered hydrofluoric acid.

For the experiments used in this work, we use membranes of 20 nm thickness. Suppl. Fig. 1 depicts the typical morphology of freestanding PAuM of 15 nm, 20 nm and 25 nm. Note that at 30 nm and beyond, a complete, non-porous gold film is observed[1]. Suppl. Fig. 1 additionally includes the equivalent pore size distribution for the 20 nm PAuM used in this work.

Tips for good transfer:

- Buffered hydrofluoric acid **without surfactant**. Otherwise the surface tension is too low to support the sample.
- After spin coating the PMMA, it is helpful to scratch along the boarders of the sample, as PMMA can flow around the edges, which can lead to a non-complete release etch in BHF.
- Use a rough surface (e.g. unpolished side of wafer) to fish the released sample from the BHF to transfer them to the rinsing baths, as the PMMA/PAuM tends to stick to smooth/plain surfaces after release.

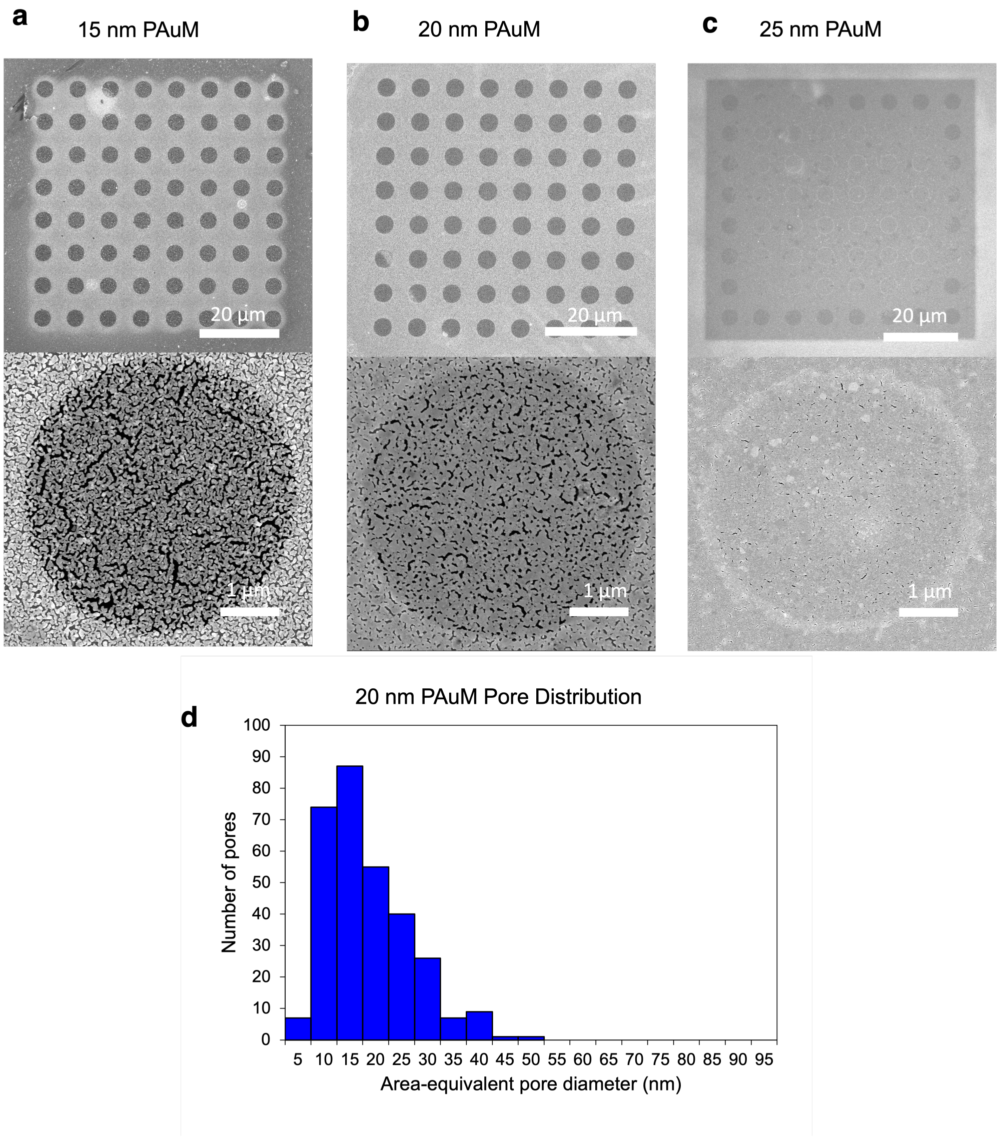

SUPPLEMENTARY FIGURE 2. **Effect of PAuM thickness on pore morphology.** PAuM of 15 nm, 20 nm and 25 nm suspended over arrays of holes of 4 µm are shown in panels **a**, **b**, and **c**, respectively. For this work, membranes with 20 nm have been used for transmission and enhancement measurements. The equivalent pore size distribution is shown for this particular type of membrane in **d**. The equivalent pore diameter is obtained by extracting the area of the pore and subsequently calculating a diameter based on a circular geometry. To obtain the pore statistics, 3 SEM images were obtained from the sample shown in **b** at higher magnification and a total of 350 pores were analyzed.

Removing PAuM from the surface:

The gold membranes can be removed from many materials, e.g. using a potassium-iodide (KI) gold etch, if the substrate material is compatible with KI etchants. Furthermore, simple peeling-off of the membrane can also be performed, especially for ceramics, where gold typically has a very low adhesion. Another method is simple scratching of the membrane if the sample allows this treatment. The latter is illustrated in Suppl. Fig. 3.

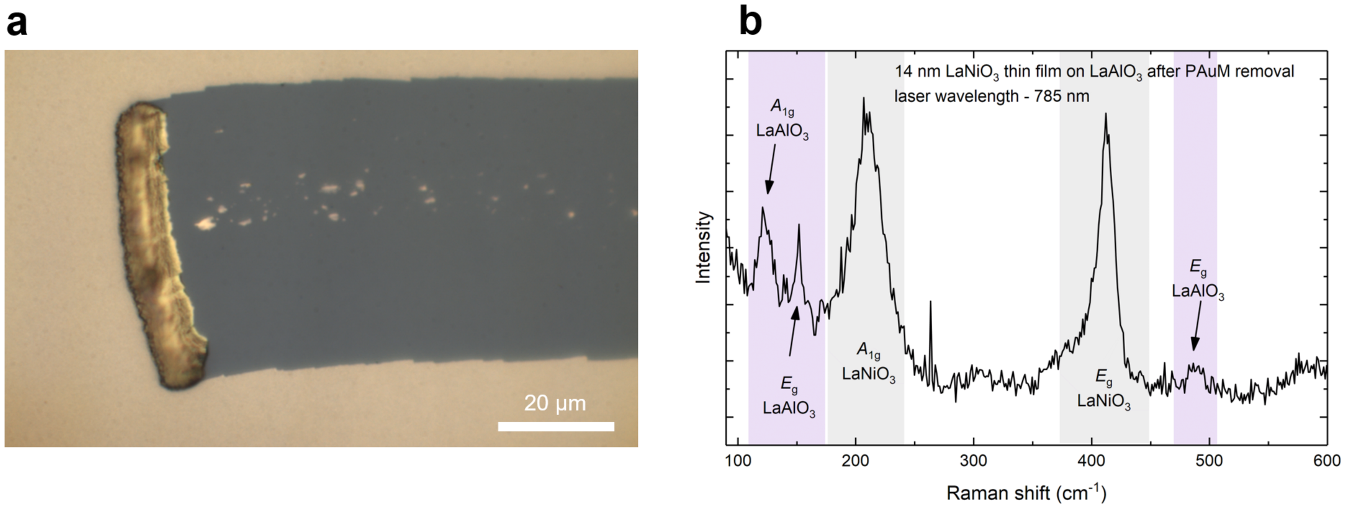

SUPPLEMENTARY FIGURE 3. **Manual removal of the PAuM of a 14 nm $LaNiO_3$ thin film.** **a** Photo of the removal of the PAuM of a $LaNiO_3$ thin film on $LaAlO_3$. **b** Raman spectrum at the position, where the PAuM has been removed. The Raman spectrum is in very good agreement with literature data for a 14 nm $LaNiO_3$ thin film[2,3].

SUPPL. NOTE 2. INTERFERENCE EFFECTS ON SURFACE ENHANCEMENT

When measuring the Raman intensity of graphene or other two-dimensional materials on stratified media, one has to take into account the effects of interference on the measured Raman intensities[4,5]. The interference arises from partial reflection and transmission of both the excitation and the Raman scattered light at the interfaces of the different media, and depends on their refractive index and thicknesses. To account for interference due to the 300 nm SiO_2 oxide layer, we apply the formalism of transfer matrices as done in Ref.[4]. In Suppl. Table 1, we list the factors by which the laser intensity (F_L) and the G- and 2D-mode intensities (F_G, F_{2D}) are modulated due to interference. The products $F_L \times F_G$ and $F_L \times F_{2D}$ indicate how the measured intensities of the G- and 2D-modes are affected by interference. The interference primarily affects our graphene reference measurements without PAuM. The 2D mode intensity for an excitation wavelength of 785 nm, for instance, is reduced to 10% of its true value. For graphene covered by PAuM, on the other hand, interference effects are negligible, see discussion in Suppl. Note 4. To account for interference, the enhancement by the PAuM for the graphene 2D mode is hence given as

$$\mathrm{Enh(2D)} = \frac{I_{\mathrm{PAuM}}(\mathrm{2D})}{I_{\mathrm{ref}}(\mathrm{2D})} \times F_{\mathrm{L}} \times F_{\mathrm{2D}}, \tag{1}$$

with the experimentally measured Raman intensities with PAuM ($I_{\mathrm{PAuM}}(\mathrm{2D})$) and without PAuM ($I_{\mathrm{ref}}(\mathrm{2D})$) from Fig. 2 of the main paper. The enhancements for the G-mode are calculated in the same way. The interference factors are shown in Suppl. Table 1. All enhancements presented in Table 1 of the main paper include interference effects and are calculated from Eq. 1.

SUPPLEMENTARY TABLE 1. List of factors to account for interference modulation of the laser intensity (F_L) and the of graphene G-mode (F_G) and 2D-mode (F_{2D}) for 300 nm SiO_2 on Si.

laser	F_L	F_G	F_{2D}	$F_L \times F_G$	$F_L \times F_{2D}$
532 nm	1.280	1.644	1.474	2.11	1.89
660 nm	1.118	0.516	0.268	0.577	0.3
785 nm	0.308	0.199	0.33	0.061	0.1

SUPPL. NOTE 3. INTERFERENCE EFFECTS ON BULK SUPPRESSION

Similar to measuring the Raman intensity of graphene placed on stratified media, interference effects due to reflection and transmission also affect the intensity of the Si Raman peak, which we use in the main paper to illustrate the effect of bulk suppression by the PAuM. To illustrate this, we numerically simulate the intensity distribution $I_1(z) = (E(z)/E_0(z))^2$ of two incoming plane waves with $\lambda = 660\,\mathrm{nm}$ (excitation, $I_0 = E_0^2$) and $\lambda = 683.2\,\mathrm{nm}$ (corresponding Si Raman peak) along the z-axis of our Si/SiO_2/air interface without graphene and PAuM in Suppl. Fig. 4a. Details of the simulation are discussed in Suppl. Note 4. We find that at the SiO_2/Si interface and further into the Si, the intensity has dropped by a factor of 5 compared to the I_0. For comparison, we calculate the intensity distribution without Si in Suppl. Fig. 4b and find an intensity drop by a factor of 1.67. This shows that we have to consider the effect of interference when quantifying bulk suppression since interference affects our reference measurements of the Si bulk Raman peak without the PAuM.

Suppl. Fig. 4c plots the ratio of the light intensities in Suppl. Fig. 4a,b as E^4. We find that at the SiO_2/Si interface and further into the bulk, the E^4 enhancement due to interference yields a factor of 0.1. This suggest that the Si Raman signal without PAuM, which we use as reference to estimate bulk Raman suppression in the main paper, should be corrected accordingly by a factor 10. The suppression of the bulk Raman signal as measured in our experimental configuration as sketched in Suppl. Fig. 4a is therefore closer to a factor of 100 as compared to a factor 10 stated in the main paper.

We do not provide an in-depth quantification of the interference effect in bulk Raman signal suppression here since this effect depends on the exact structure of a sample investigated and other experimental parameters (refractive index of the material(s)/layers, numerical aperture of the objective, confocality of the experimental setup,..). Providing exact and interference corrected values for bulk suppression for our model geometry will therefore bring little insight for the general use case. We therefore provide the experimental values in the order of a factor 10 in the main paper as a lower bound for bulk suppression, and stress that we expect the bulk suppression to be higher in typical use cases.

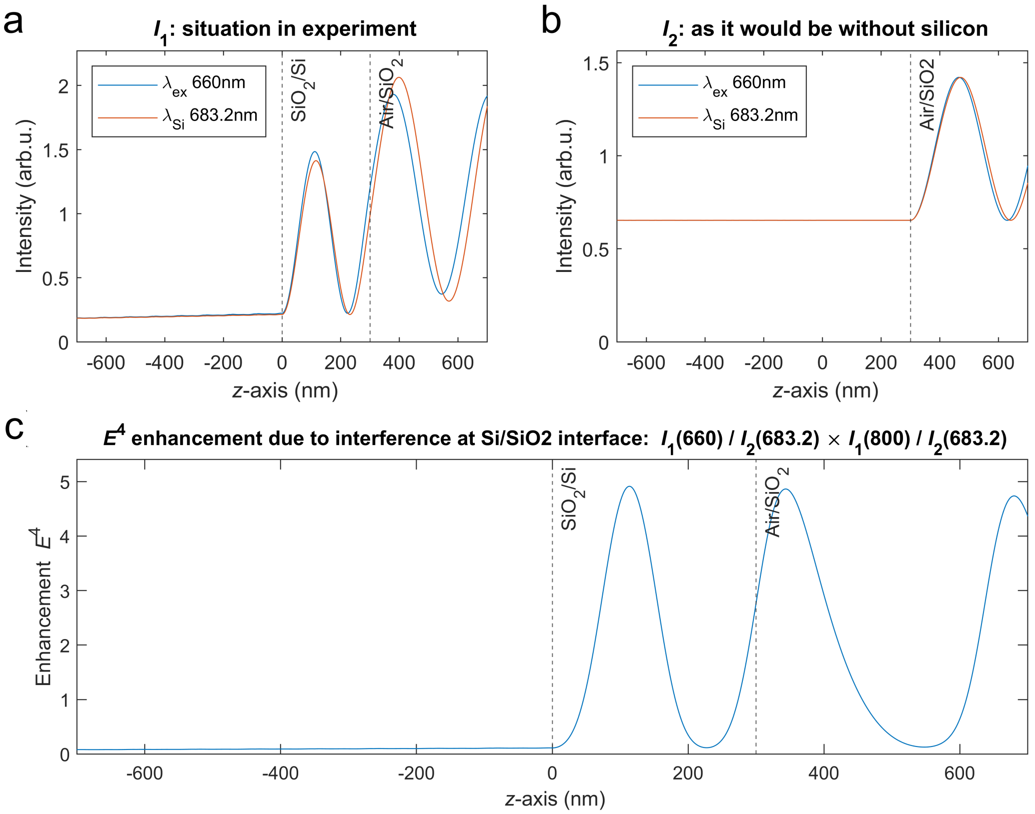

SUPPLEMENTARY FIGURE 4. **Interference effects on bulk Raman signal suppression.** **a** Intensity modulation due to interference at multiple interfaces as in the experiment **b** Intensity modulation due to interference if the Si were missing. **c** E^4 enhancement due to interference comparing the situation as found in the experiment in **a** and without Si **b**.

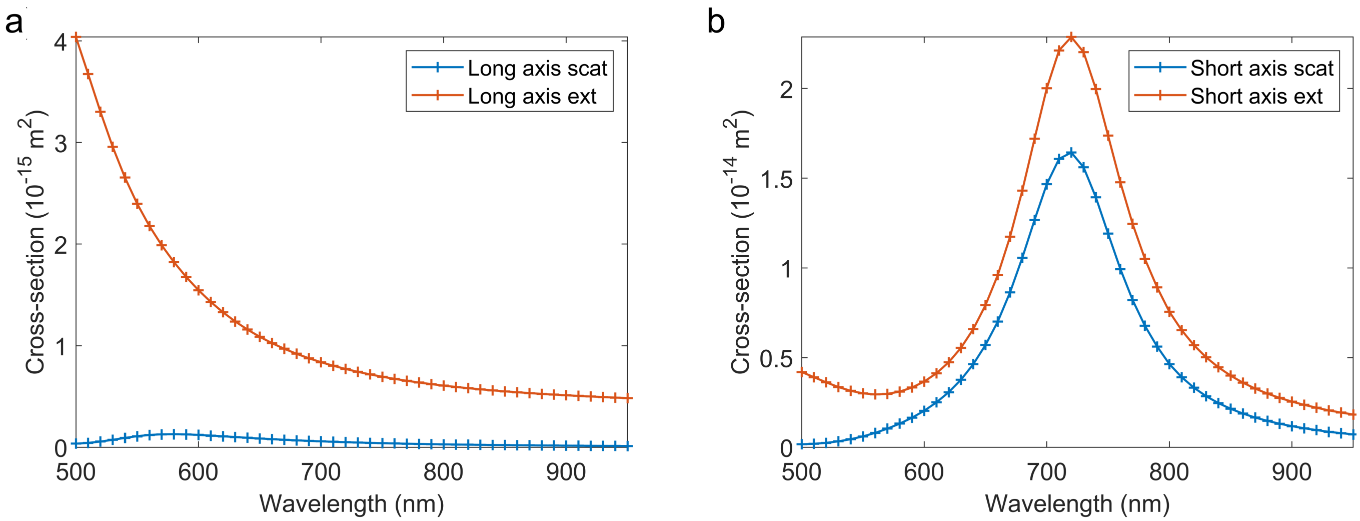

SUPPLEMENTARY FIGURE 5. **Resonance simulation of model pore.** Extinction and scattering cross section of 68 nm x 10 nm slot antenna excited along long **a** and short axis **b**, respectively. The resonance at around 720 nm is responsible for the enhancement of the graphene Raman scattering in the situation exemplified here.

SUPPL. NOTE 4. NUMERICAL FINITE ELEMENT SIMULATIONS

The aspect ratio of our prototypical nanoslot, see Methods in the main paper, is chosen to yield 720 nm resonance wavelength, as it lies between the excitation wavelength in the experiment and the corresponding Raman 2D line of graphene. We use a 5 nm radius for the rectangular slot edge. The radius in the direction of stacking is estimated from SEM images of the membranes. The shape can be seen in the xz-cut of Suppl. Fig. 6c.

To estimate the Raman enhancement, we computed the near fields for the excitation wavelength $\lambda_{\text{excitation}}$ as well as for the Raman peak wavelength λ_{Raman}. If not stated differently, we used $\lambda_{\text{excitation}} = 660\,\text{nm}$ and $\lambda_{\text{Raman}} = 800\,\text{nm}$ (corresponding to the 2D line of graphene). Then we computed the Raman enhancement E^4 according to

$$E^4 = \left(\frac{E(\lambda_{\text{excitation}})}{E_0(\lambda_{\text{excitation}})}\right)^2 \cdot \left(\frac{E(\lambda_{\text{Raman}})}{E_0(\lambda_{\text{Raman}})}\right)^2, \tag{2}$$

where E_0 denote the fields, when no metallic film is present. Heatmaps and a depth scan of E^4 can be seen in Suppl. Fig. 6.

In our experiment, we have probed the Raman signal of graphene for different SiO_2 layer thicknesses. The substrate consisted of a SiO_2 on a Si wafer and the interface to Si leads to reflections that might alter the effective Raman enhancement. To study the effect of the Si substrate at about 300 nm below the gold-SiO_2 interface, we computed the same Raman enhancement including a silicon substrate. We found that these reflections have a negligible impact on the Raman signal when the gold membrane is present (Suppl. Fig. 7). For sample areas without a gold membrane, we computed that the enhancement with silicon reaches about 40 % of that without silicon only, due to interference

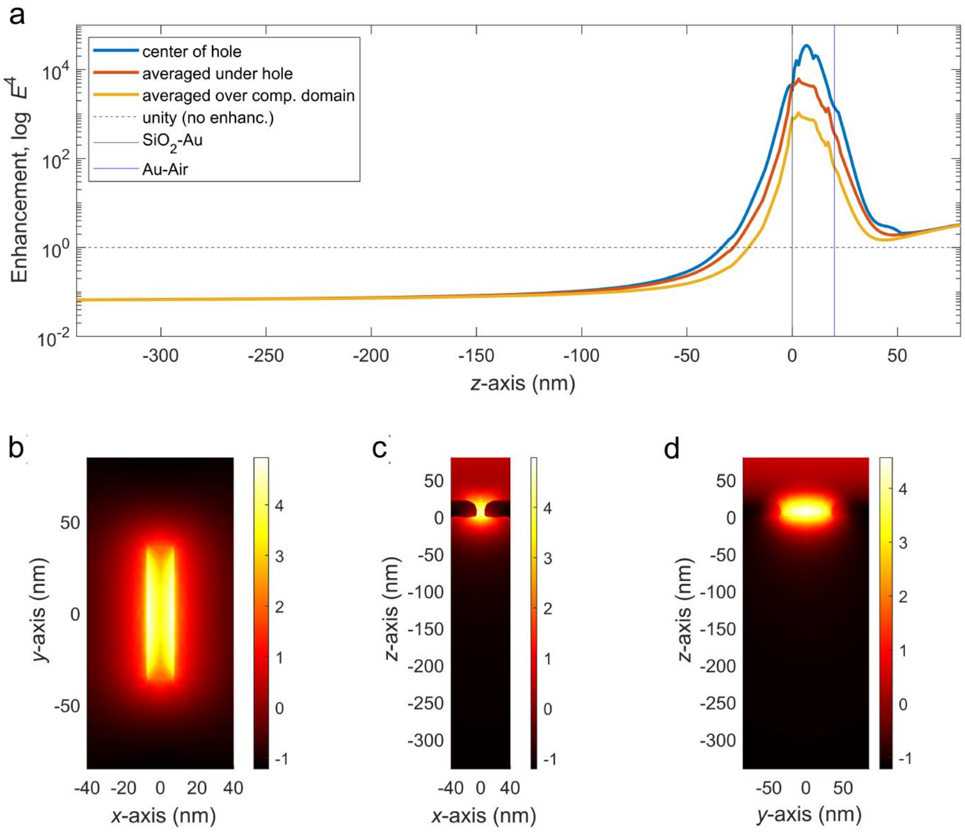

SUPPLEMENTARY FIGURE 6. E^4 **enhancement (log scale) for a 22 nm gold membrane with a 10 nm x 68 nm slot on** SiO_2**. a** Enhancement as a function of z-position tracked along the center of the slot antenna or averaged over areas (just below the slot or over the complete computational domain). The exponential decay of the enhancement below the gold pore is evident. **b** xy-cut at gold-SiO_2 interface, **c** and **d**: cuts along xz- and yz-planes, respectively. The color scales in **b-d** are also log scales of E^4.

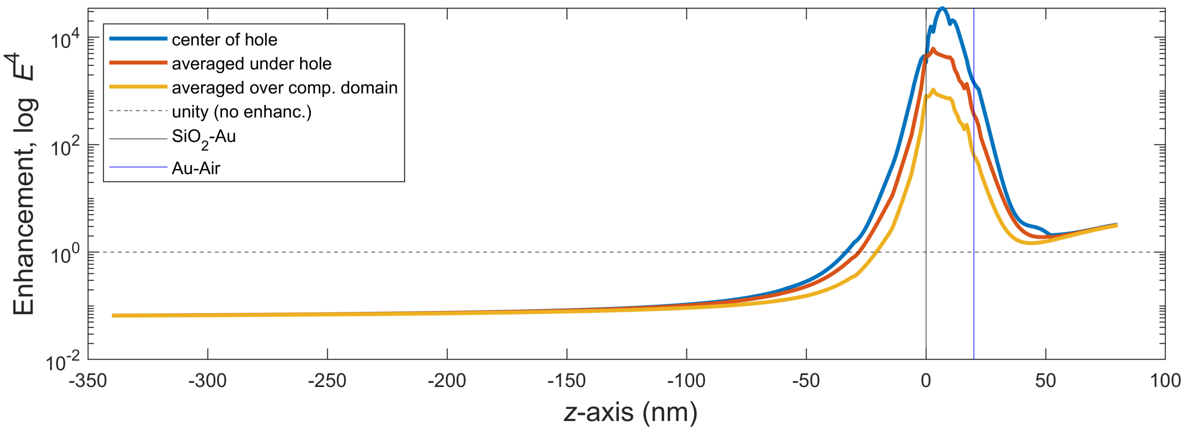

SUPPLEMENTARY FIGURE 7. E^4 **enhancement (log scale) for arrangement with 300 nm** $\mathbf{SiO_2}$ **on a silicon substrate**. Enhancement is shown as a function of z-position tracked along the center of the slot antenna or averaged over areas (just below the slot or over the complete computational domain). The exponential decay of the enhancement below the gold pore is evident.

effects, which is in line with Ref. [5]. We discuss the implications of interference on bulk Raman signal suppression in detail in Suppl. Note 3.

We also investigated other slot aspect ratios. As mentioned above, smaller slots yield higher enhancements and steeper decays into the substrate. However, an exponential decay appears to be a general feature of resonant pores, whereas off-resonant pores yield significantly flatter decays into the substrate with small enhancement factors. Suppl. Fig. 8 shows results for a 30 nm x 80 nm slot. This slot also has a resonance wavelength at 720 nm. For this configuration we also studied other Raman shifts. All E^4 enhancements depth scans show a very similar decay behavior. See Suppl. Note 5 for a detailed discussion of the depth-dependence of surface Raman enhancement by PAuM.

We also studied the impact of higher refractive index substrates. In this case, resonance positions of the pores will shift to higher wavelength. To compare pore performance for higher index substrates to those before, we thus adjusted the pore size to reach a similar resonance frequency. Suppl. Fig. 9 depicts an example with a refractive index of 2.5 of the substrate. The respective slot has a smaller aspect ratio with 36 nm x 10 nm resulting in a resonance at around 710 nm. Again, we find an exponential decay of the enhancement factor E^4 (Suppl. Fig. 9). The overall E^4 enhancement is smaller which we attribute to an *effectively* larger pore size of the slot due to the higher index of the substrate, i.e. even smaller pores will yield higher E^4 enhancements with this refractive index.

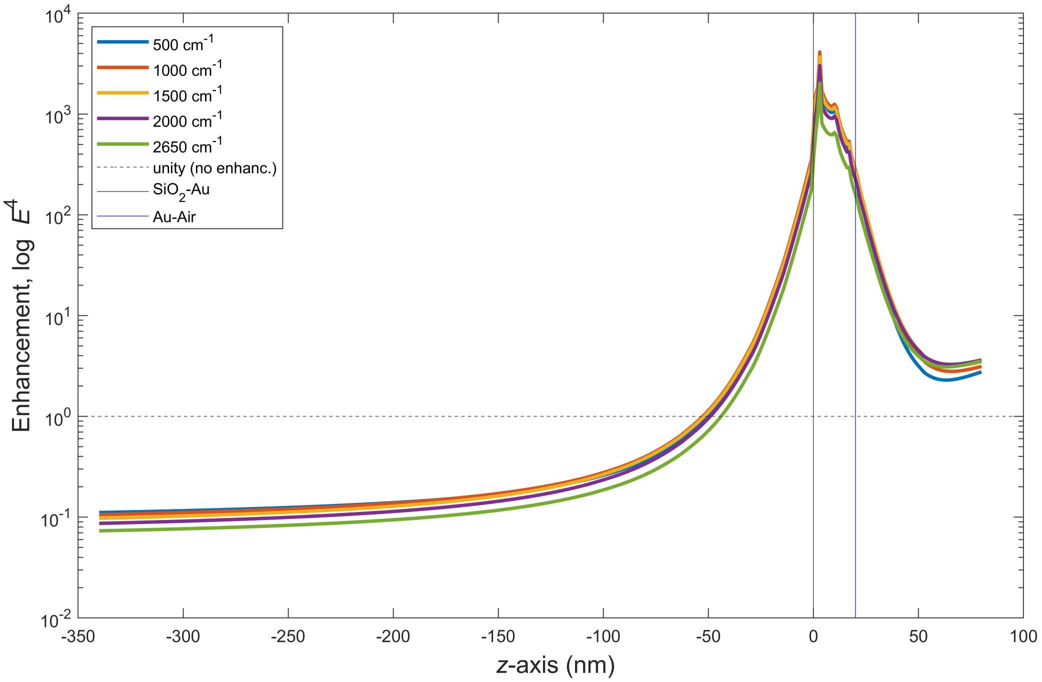

SUPPLEMENTARY FIGURE 8. E^4 **enhancement (log scale) for a 30 nm x 80 nm slot and various Raman shifts as a function of** z**-position.** The enhancement is averaged over the area just below the slot. The exponential decay of the enhancement below the gold pore is evident. The $1500\,\mathrm{cm}^{-1}$ Raman emission curve relates to 732.5 nm which is close to the antenna resonance.

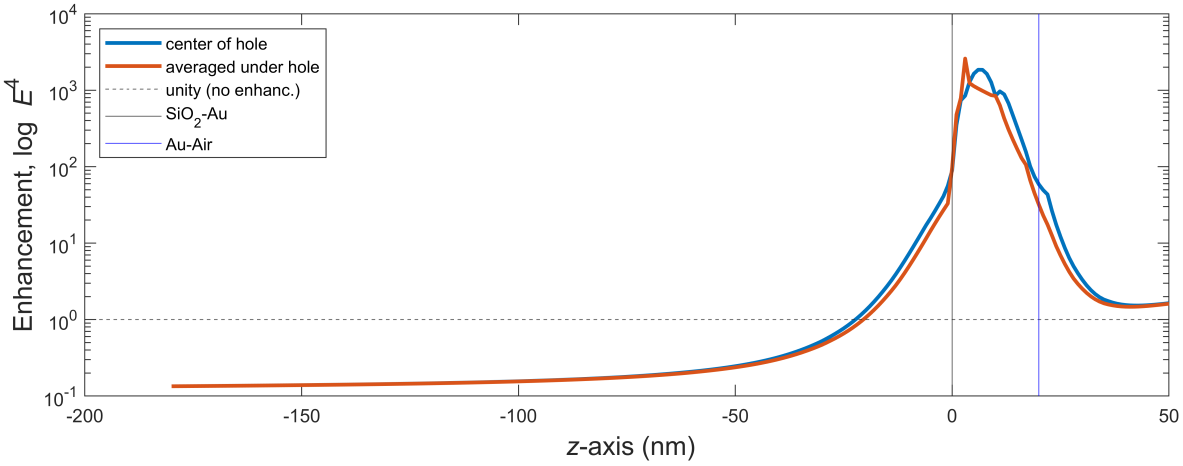

SUPPLEMENTARY FIGURE 9. E^4 **enhancement (log scale) for a 22 nm gold membrane with a 10 nm x 36 nm slot on a medium with a refractive index of 2.5 as a function of** z**-position**. The enhancement is tracked along the center of the slot antenna or averaged below the slot. The resonance wavelength is around 710 nm. As before, an exponential decay of the enhancement below the gold pore is evident (note the shorter z-axis compared to figures before).

SUPPL. NOTE 5. DEPTH-DEPENDENCE OF SURFACE RAMAN ENHANCEMENT

In Suppl. Fig. 10 we compare the simulated Raman enhancement as a function of depth z for different plasmonic antennas for the 2D Raman mode at 800 nm for an excitation wavelength of 660 nm. The maximum enhancement at $z = 0$ nm is normalized to 1 for each antenna to enable a comparison between the different antennas. The grey dots mark average enhancement over the commputational domain for the 10 nm x 68 nm slot antenna extracted from Suppl. Fig. 6 (yellow curve). This area includes the area around the slots, which is about 15 times larger than the slot alone. The data is fitted well with a simple exponential decay (black curve) with a decay constant of $\tau = 1$ nm, see main paper. The black arrow indicates the depth up to which 90 % of the total enhancement occurs. To validate that both our simulation and experimental results on the depth-dependence of surface Raman enhancement are reasonable, we compare it to tip-enhanced Raman spectroscopy (TERS) measurements[6] as the red curve in Suppl. Fig. 10.

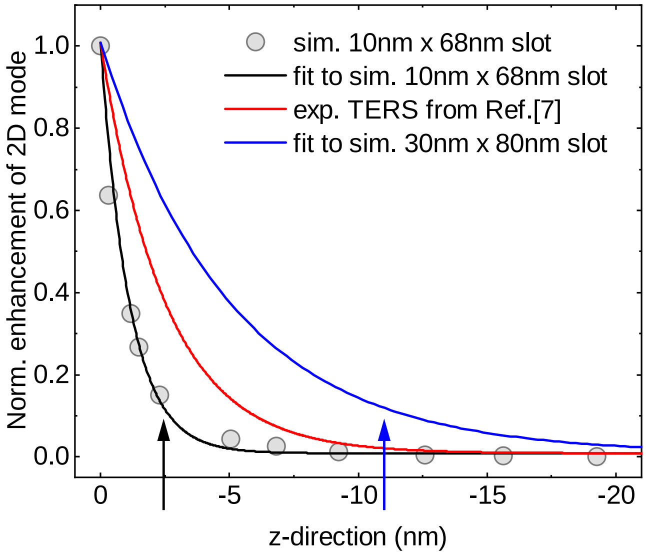

SUPPLEMENTARY FIGURE 10. **Simulated enhancement as a function of depth for different plasmonic antennae.** Depth-dependence of the surface-enhancement for a 10 nm x 68 nm plasmonic slot antenna (dots and black line) as in the min paper, a 30 nm x 80 nm plasmonic slot antenna (blue) and a golden pyramid used in a tip-enhanced Raman spectroscopy experiment (red). Arrow indicate the depth up to which 90% of the total enhancement occurs.

In these measurements, a sharp Au pyramid is placed directly on top of a carbon nanostructure (confined carbyne) to enhance the Raman signal. The red curve in Suppl. Fig. 6 shows how the enhanced Raman signal drops when the tip is retracted, whereby an increasing tip-sample distance corresponds to an increase in depth / z-direction. For the TERS-tip, the enhancement drops exponentially with the tip-sample distance ($\tau = 2.5$ nm). We conclude that the exponential decay of the Raman enhancement with distance from the plasmonic hotspost (nanoslot/tip apex) is a common feature of plasmon-enhanced Raman scattering, which is in agreement with literature[7].

The rate at which the Raman enhancement drops as a function of depth in the material may vary for different nanoslots. To illustrate this, we plot the normalized 2D mode enhancement vs. z for a 30 nm x 80 nm nanoslot (see Suppl. Fig. 8) as a blue curve in Suppl. Fig. 10. The drop in enhancement (τ =5 nm) is not as sharp as for the other slot antenna (black) and 90 % of the total enhancement occurs within the first 11.5 nm as indicated by the blue arrow. The parameter that distinguishes these two nanoslots is the quality factor of the plasmonic resonance. see Suppl. Note 4. The 10 nm x 68 nm nanoslot antenna features a comparably high quality factor, which is associated with a high peak enhancement and a sharp drop of the enhancement with distance from the slot. The 30 nm x 80 nm nanoslot antenna, on the other hands, comes with a smaller quality factor, which in turns features a 7-fold weaker

maximum enhancement and, by comparison, a smooth decrease of the enhancement with distance from the nanoslot antenna.

This suggest that the most surface-sensitive Raman signal is given by hotspots/nanopores that show the highest Raman intensity in the experiment. Locations that show a weaker Raman enhancement capture Raman features from deeper inside the material and are hence less surface sensitive than the high-enhancement nanopores.

SUPPL. NOTE 6. GRAPHENE 2D-MODE ENHANCEMENT WITH SPACERS

In Suppl. Fig. 11a we show a line scan of the graphene 2D-mode enhancement across the edge of a PAuM that is separated from the graphene by a 5 nm SiO_2 spacer for 532 nm (green squares) and 660 nm excitation (red circles), sample preparation see Methods section. For both excitation wavelengths, the intensity is normalized to the average integrated peak intensities recorded without the PAuM (x >8 µm).

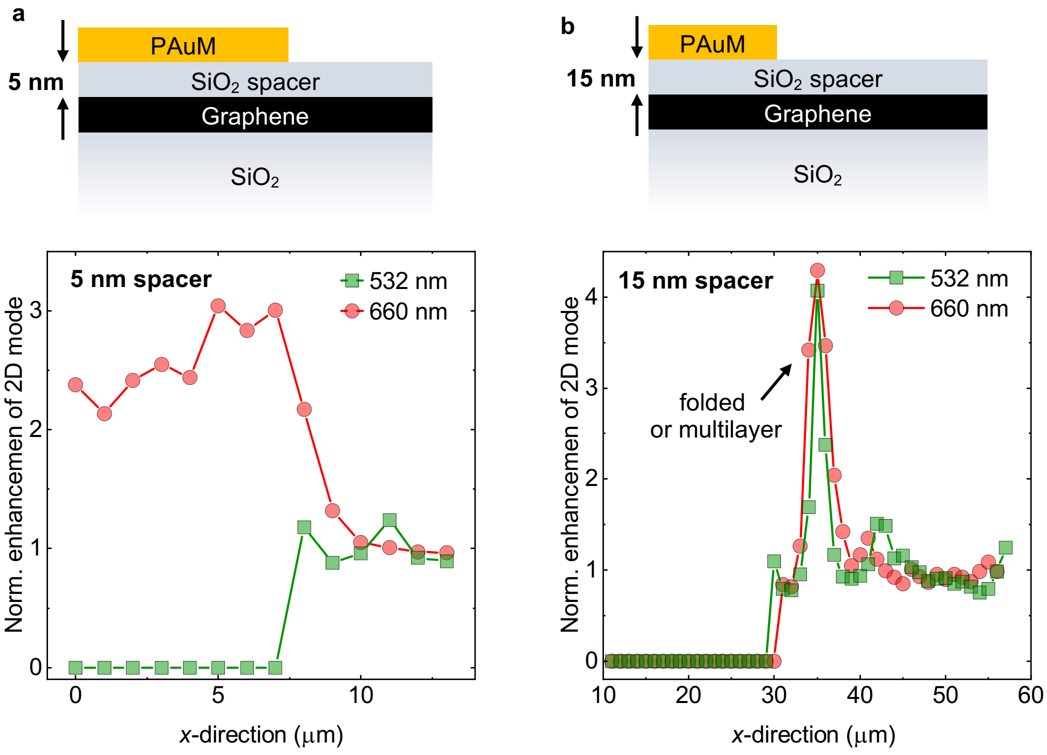

SUPPLEMENTARY FIGURE 11. **Graphene 2D-mode enhancement with Spacers.** The linescans depict the 2D-Raman mode enhancement normalized to the average integrated 2D-mode intensity without PAuM for **a** 5 nm and **b** 15 nm SiO_2 spacer between PAuM and the graphene for 532 nm and 660 nm excitation.

With the PAuM on top, we find a maximum enhancement of 3 for 660 nm, while no 2D mode is measureable for 532 nm. This data can be understood as the interplay between a residual enhancement of the 2D mode by the pores in the PAuM and the signal suppression for 660 nm, while the suppression dominates for 532 nm. For the 15 nm SiO_2 spacer, Suppl. Fig. 11b, no 2D-mode signal of graphene is detected for both excitation wavelengths. This means this for this spacer thickness, the graphene is too far away from the PAuM to be enhanced. The enhancement values obtained here experimentally for 660 nm are shown in Fig. 3b of the main paper.

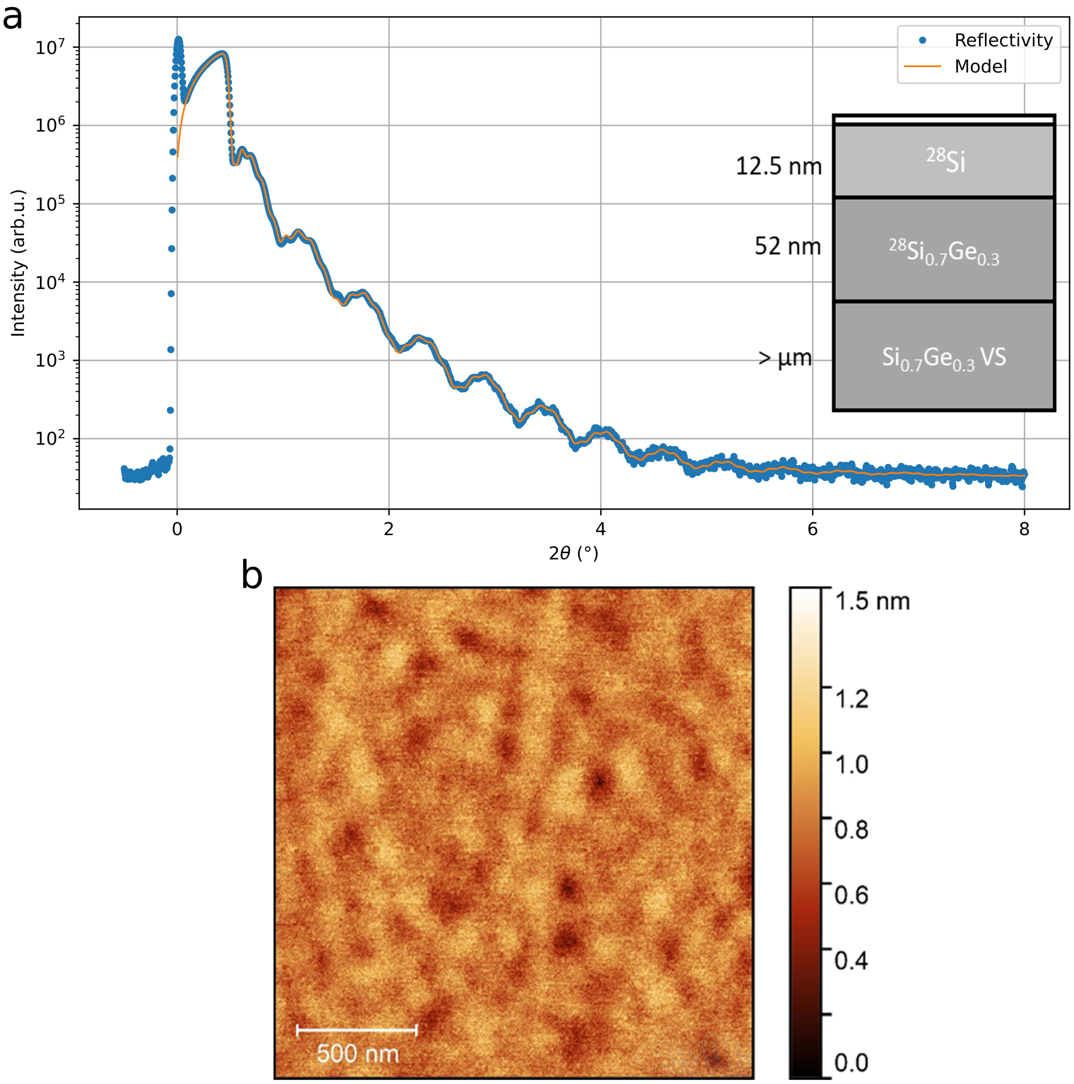

SUPPLEMENTARY FIGURE 12. **Characterization of the $^{28}Si/^{28}Si^{76}_{0.7}Ge_{0.3}$ heterostructures.** **a** X-ray reflectivity profile with a model fit consisting of a SiGe virtual substrate, a layer for damaged SiGe for chemo-mechanical polishing, and the MBE grown heterostructure (SiGe/Si) with a thin SiO_2 layer and air, as sketch in the Figure inset. **b** AFM topography image of the strained ^{28}Si layer.

Layer	Thickness (nm)	Roughness (nm)	Density (g cm^{-3})	Composition
Air	inf	0.0000	0.001	$N_{0.78}O_{0.21}$
SiO_2	2.1837	0.1730	2.326	SiO_2
Si	12.4890	3.0912	2.330	Si
SiGe	52.3322	0.4108	3.122	$Si_{0.76}Ge_{0.24}$
SiGe (CMP)	1.0803	1.2566	3.426	$Si_{0.67}Ge_{0.33}$
SiGe (VS)	inf	0.0004	3.332	$Si_{0.70}Ge_{0.30}$

SUPPLEMENTARY TABLE 2. Results for the best fit solution to the six layer model applied on the X-ray reflectivity data in Suppl. Fig. 12a.

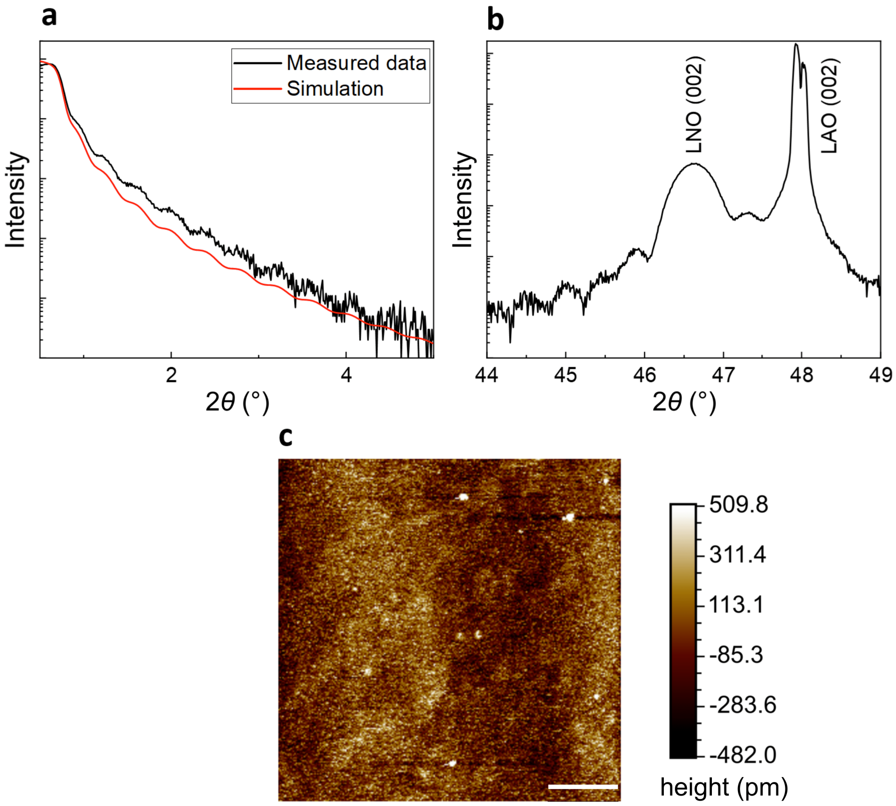

SUPPLEMENTARY FIGURE 13. **Structural characterization of the $LaNiO_3$ thin film grown on $LaAlO_3$.** **a** Measured and simulated X-ray reflectometry profiles for the deposited $LaNiO_3$ film with an extracted film thickness of 20.8 nm according to the Kiessig fringe periodicity. **b** Symmetric $\Theta - 2\Theta$ X-ray diffraction scan around the $LaAlO_3$ 002 peak. The presence of a single $LaNiO_3$ 002 reflection with clear Laue oscillations indicates high crystalline quality of the $LaNiO_3$ film. **c** AFM topography scan in tapping mode, showing the atomically flat $LaNiO_3$ film. The scale bar in **c** is 1 µm.

SUPPL. NOTE 7. PAuM-ENHANCED RAMAN SPECTROSCOPY ON $LaNiO_3$ THIN FILMS

Suppl. Fig. 14a displays a PAuM-enhanced Raman spectrum of the $LaNiO_3$ thin on $LaAlO_3$. With a laser excitation wavelength of 785 nm the spectrum is measured under comparable conditions as in Fig. 5b of the main text, yet with higher spectral resolution. The higher spectral resolution brings out the splitting of the E_g mode even further.

Suppl. Fig. 14b depicts the Raman spectra with and without PAuM-enhancement using a laser with a wavelength of 660 nm. Similarily to the case with 785 nm-excitation, the substrate signal is suppressed and the signature of the $LaNiO_3$ thin film exhibits a prominent shoulder at $\approx$380 cm^{-1} indicating the E_g-mode splitting. However, the signal is less well-defined in accordance with the better surface-signal enhancement for 785 nm excitation.

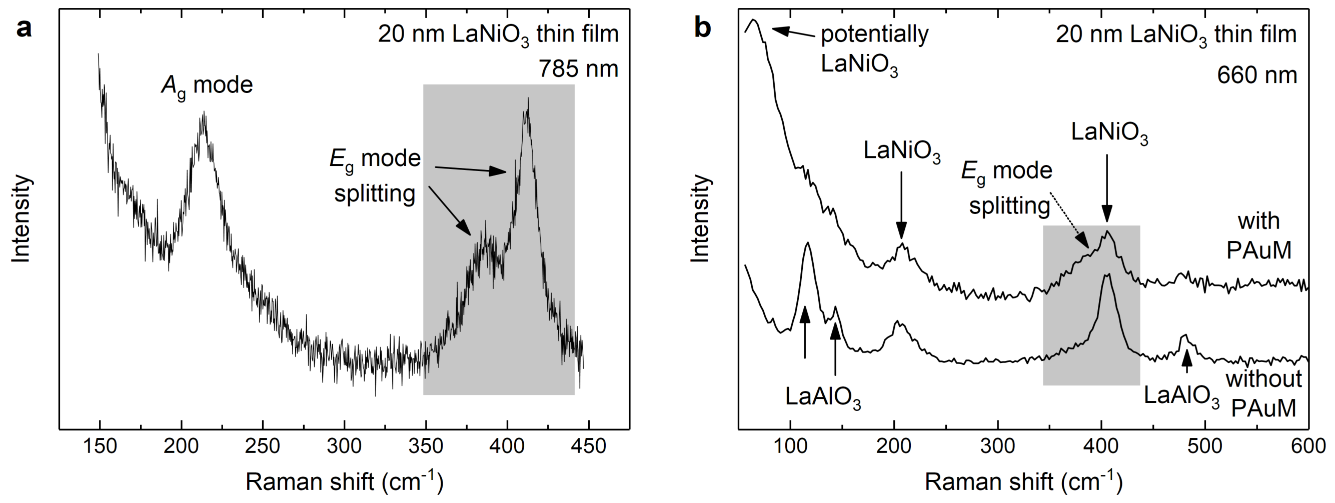

SUPPLEMENTARY FIGURE 14. **Wavelength-dependent Raman measurement of $LaNiO_3$ thin on $LaAlO_3$. a** High-reslution PAuM-enhanced Raman spectrum of the $LaNiO_3$ thin on $LaAlO_3$ using an excitation wavelength of 785 nm. **b** The Raman spectrum of a $LaNiO_3$ thin film on $LaAlO_3$ with PAuM and without PAuM are depicted under 660 nm-excitation.

Suppl. Fig. 15 shows the Raman spectra of **a** a 14 nm and **b** a 7 nm $LaNiO_3$ thin film on $LaAlO_3$ using an excitation wavelength of 785 nm with PAuM and without PAuM. The spectra with PAuM membrane show the very similar spectral signatures with a clear E_g-mode splitting such as for the 20-nm thin film, see main paper. The fact that the Raman signal is independent of the film thickness confirms that it arises primarily from the film's surface.

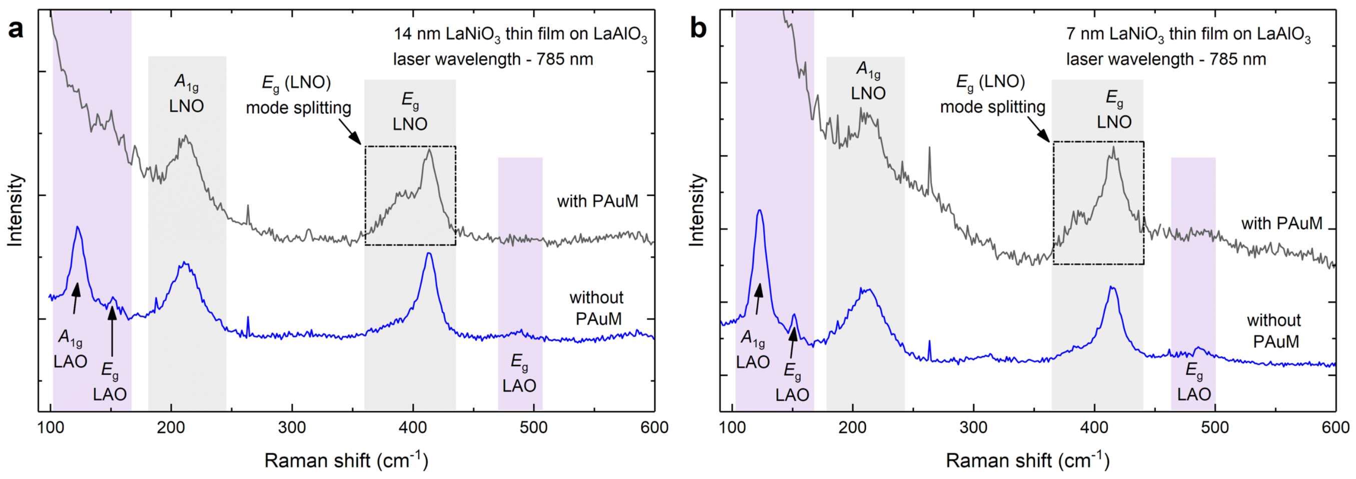

SUPPLEMENTARY FIGURE 15. **Thickness-dependent Raman measurement of $LaNiO_3$ thin on $LaAlO_3$** Raman spectra of **a** a 14 nm and **b** a 7 nm $LaNiO_3$ thin film on $LaAlO_3$ using an excitation wavelength of 785 nm with PAuM and without PAuM.

SUPPL. NOTE 8. SURFACE-SENSITIVE RAMAN SCATTERING AT LOW TEMPERATURES

To demonstrate that surface-sensitive Raman scattering can also be performed at cryogenic temperature, we mounted the sample presented in Fig. 2 of the main paper in a Janis SVT-200 Option 5 cryostat with high optical access and attached a PI Acton 2150 spectrometer. The objective (NA 0.81) was inside the cryostat and we used an excitation wavelength of 700 nm, power 0.6 mW, with integration times of 10 s.

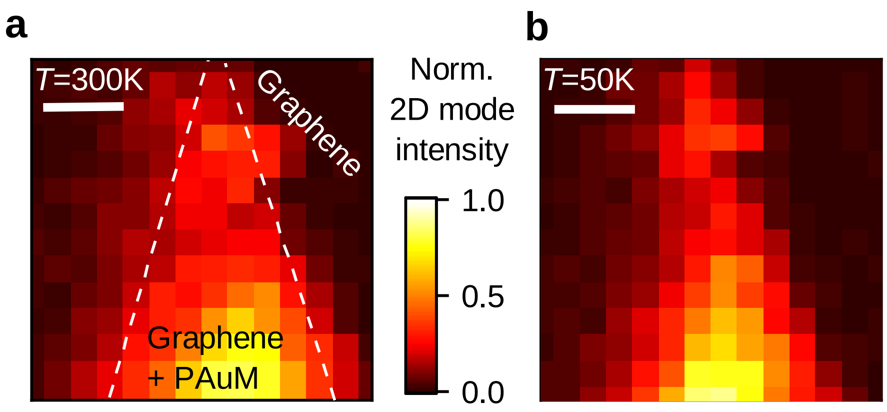

SUPPLEMENTARY FIGURE 16. **Room temperature vs. low-temperature surface-sensitive Raman scattering on PAuM/Graphene/SiO_2/Si** - Spatial Raman maps of the integrated graphene 2D-mode intensity from a cutout of the sample presented in Fig. 2 of the main paper for **a** room-temperature (300 K) and **b** cryogenic temperature (50 K) and 700 nm excitation. The scale bar is 2 µm for both maps.

Suppl. Fig. 16a,b show Raman maps of the integrated graphene 2D-mode intensity for a small cut of the the sample presented in Fig. 2 of the main paper, centered on a triangular graphene/PAuM overlap region for temperatures of 300 K and 50 K, respectively. We observe an enhancement of the graphene Raman signal where the graphene iscovered by the PAuM with comparable intensities for both temperatures. This confirms that surface-sensitive Raman scattering using PAuM also can be performed at cryogenic temperatures.

SUPPL. NOTE 9. FEASIBILITY OF USING PAUM TO PROBE ROUGH SURFACES

Characterizing rough surfaces with surface-sensitive and bulk-suppressed Raman scattering by PAuM, i.e., for investigating battery materials, is in principle possible as well. PAuM are stable enough to span over holes of several µm in diameter without breaking, essentially forming a drum-like structure. Furthermore, they are flexible on a macroscopic scale, e.g., the transferred PAuM on polycarbonate track-etched (1) membranes in the Supporting Information of Wyss et al.[1]. That membrane was transferred by heat-induced bonding and peeling of the PCTE-PAuM from the support wafer.

Depending on the specifics of the roughness of the surface of interest, the PAuM will be in contact with a certain number of sites per surface area of the underlying substrate. This corresponds to a contact area A_c that might be described with some ratio r to the overall area A_{tot} as $r = A_c/A_{tot}$. Then, the likelihood of detecting an enhanced Raman signal from the surface per illuminated and observed surface area is simply weighted with this ratio r. The suppression of bulk Raman signals is independent of the surface roughness and the contact area between the material surface and the PAuM. Characterizing rough surfaces with surface-sensitive and bulk-suppressed Raman scattering will require dedicated process development and optimization of the transfer of PAuM onto rough surfaces to achieve optimal results.

REFERENCES

SUPPLEMENTARY REFERENCES

1. Wyss, R. M. et al., Freestanding and permeable nanoporous gold membranes for surface-enhanced Raman scattering, *ACS Appl. Mater. Interfaces* **14**, 14, 16558 (2022).
2. Weber, M. C. et al., Multiple strain-induced phase transitions in $LaNiO_3$ thin films, *Phys. Rev. B* **94**, 1, 014118 (2016).
3. Schober, A. et al., Vibrational properties of $LaNiO_3$ films in the ultrathin regime, *APL Mater.* **8**, 6, 061102 (2020).
4. Narula, R., Pankin, R. and Reich, S., Absolute Raman matrix elements of graphene and graphite, *Phys. Rev. B* **82**, 4, 045418 (2010).
5. Yoon, D. et al., Interference effect on Raman spectrum of graphene on SiO_2/Si, *Phys. Rev. B* **80**, 125422 (2009).
6. Heeg, S., Shi, L., Poulikakos, L. V., Pichler, T. and Novotny, L., Carbon Nanotube Chirality Determines Properties of Encapsulated Linear Carbon Chain, *Nano Lett.* **18**, 9, 5426 (2018).
7. Novotny, L. and Hecht, B., *Principles of Nano-Optics*, Cambridge University Press, 2006.
